# Sex-Specific Differences in Pre-Stroke Characteristics Reveal Vulnerability of Elderly Women

**DOI:** 10.3390/jpm12030344

**Published:** 2022-02-24

**Authors:** Carolin Hoyer, Jan Schlenker, Vesile Sandikci, Anne Ebert, Matthias Wittayer, Michael Platten, Kristina Szabo

**Affiliations:** 1 Department of Neurology, Medical Faculty Mannheim, Mannheim Center for Translational Neuroscience (MCTN), Heidelberg University, 68167 Mannheim, Germany; carolin.hoyer@umm.de (C.H.); j.schlenker@stud.uni-heidelberg.de (J.S.); vesile.sandikci@umm.de (V.S.); anne.ebert@umm.de (A.E.); matthias.wittayer@umm.de (M.W.); michael.platten@umm.de (M.P.); 2 CCU Healthy Brain, Competence Network Preventive Medicine Baden-Württemberg, 68167 Mannheim, Germany

**Keywords:** stroke, sex, risk factors, pre-stroke disability

## Abstract

While the sexually dimorphic character of ischemic stroke has been acknowledged along several dimensions, age-specific sex disparities regarding pre-stroke characteristics in particular have received comparatively little attention. This study aimed to identify age-dependent associations between sex and risk factors, premorbidity, and living situation in patients with ischemic stroke to foster the continuing development of dedicated preventative strategies. In a retrospective single-center study, data of patients with acute ischemic stroke (AIS) admitted to the Department of Neurology, University Hospital Mannheim, Germany, between June 2004–June 2020 were included; AIS frequency, vascular risk factors, premorbidity, living situation, and stroke etiology were analyzed across sexes and different age spectra. From a total of 11,003 patients included in the study, 44.1% were female. Women aged >70–≤90 years showed a pronounced increase in stroke frequency, lived alone significantly more frequently, and had a significantly higher degree of pre-stroke disability than men; however, only hypertension and atrial fibrillation were more prevalent in women in this age segment. The seventh and eighth decades are a critical time in which the pre-stroke risk profile changes resulting in an increase in stroke morbidity in women. This emphasizes the relevance of and need for an approach to stroke prevention that is both targeted and integrative.

## 1. Introduction

As a leading cause of long-term disability, stroke generates a substantial medical and socio-economic load [1]. Even though age-adjusted mortality rates from the disease have decreased considerably over the last 30 years, stroke incidence has remained largely unchanged due to aging and global population growth [2]. In the context of demographic change, the burden of stroke is expected to distinctly rise in women as they are going to substantially outnumber men in the coming decades [3,4]. Age, however, is not the only factor contributing to disparities between men and women as regards stroke, which is now increasingly recognized as a sexually dimorphic disorder. This dimorphism manifests in different domains such as clinical presentation, treatment, and outcome [5,6]. There are several risk factors unique to women, such as pregnancy-associated hypertension and complications as well as oophorectomy [7]. However, modifiable risk factors and socio-behavioral aspects impact stroke etiology and pathomechanisms to a more relevant extent [8]. Hence, they represent central targets for prevention and have accordingly received increasing attention in the context of investigations focusing on sex-related differences [9]. There is, however, a relative paucity of, and need for research considering different domains of sex-specific pre-stroke morbidity and functioning across the lifespan, which has been voiced to require a priori consideration in future investigations of acute stroke [10].

We thus set out to identify age-dependent associations between sex and risk factors, premorbidity, and living situation in patients with ischemic stroke to identify areas of need for future targeted preventative strategies.

## 2. Materials and Methods

We analyzed data from the Comprehensive Stroke Center database of the Department of Neurology, University Hospital Mannheim, Germany. The study was approved by the local ethics committee, consent was waived due to the retrospective design. The stroke database established at the Neurology Department in Mannheim in 2004 as a Microsoft Access database management system contains at present detailed information about more than 20,000 stroke patients. Each dataset is comprised of close to 100 variables, including demographic data, medical history, hospital admission details, functional scores (NIHSS, Barthel, modified Rankin Scale) at different time points, diagnosis, stroke type, therapeutic interventions, time course, and details of neuroimaging.

Patients with a diagnosis of acute ischemic stroke (AIS) admitted from 1 June 2004 to 30 June 2020 were included. Sex differences were compared in five distinct age categories (≤30 years; >30 and ≤50 years; >50 and ≤70 years; >70 and ≤90 years; and >90 years) regarding the frequency of AIS, pre-stroke disability assessed by the modified Rankin scale score, living situation, risk factors (hypertension, type 2 diabetes mellitus, hyperlipidemia, smoking), and the presence of cerebral and cardiac vascular disease premorbidity. Stroke etiology utilizing a modification of the categories proposed by Amarenco et al. [11] (small vessel disease, atherosclerosis, cardiac source, embolic stroke of unknown source, other, unidentified cause) was also recorded.

Statistical analysis was performed using IBM SPSS Statistics Version 27. Qualitative variables were compared using chi² test or Fisher’s exact test, depending on expected cell frequencies. Group comparisons were analyzed by Mann-Whitney U test. Missing data were excluded from the analysis. Sporadically, there were missing values in a few variables. Skewing of the results is highly unlikely due to the extremely small number of missing values. Statistical significance is indicated by *p* values of <0.05, odds ratios are given when applicable.

## 3. Results

A total of 11,003 patients were included in the study. The mean age of patients was 71.60 ± 13.30 years (range 10–105), 4857 patients (44.1%) were female.

**Figure 1:**
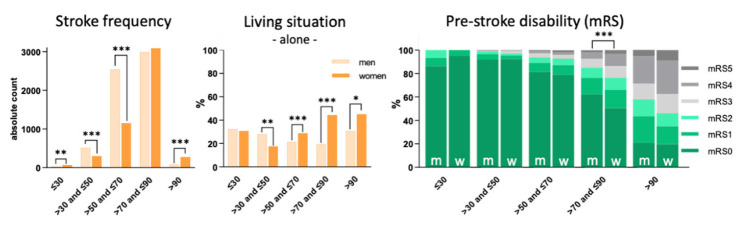
Absolute counts of acute ischemic stroke (**left**), relative frequencies of living alone (**middle**) and pre-stroke disability (**right**) in men and women across the age spectrum. mRS = modified Rankin scale, m = men, w = women; *: *p* < 0.05, **: *p* < 0.01, ***: *p* < 0.001.

Frequency of AIS. AIS frequency between men and women across the lifespan differed in all groups except for the >70–≤90 years range (≤30 years: chi² = 10.80, *p* = 0.001; >30–≤50 years: chi² = 58.82, *p* < 0.001; >50–≤70 years: chi² = 524.46, *p* < 0.001; >90 years: chi² = 105.93, *p* < 0.001). At either end of the age spectrum, absolute counts of stroke were higher in women than in men. Moreover, a pronounced increase of stroke frequency in women of ages >70–≤90 years was observed (Figure 1, left).

### 3.1. Living Situation

We identified a significant association between living situation and sex in age groups >30–≤50 years (chi² = 11.02, *p* = 0.001, odds ratio (OR) = 0.54, confidence interval (CI): 0.38–0.78), >50–≤70 years (chi² = 22.83, *p* < 0.001, OR = 1.48, CI: 1.26–1.74), >70–≤90 years (chi² = 419.86, *p* < 0.001, OR = 3.29, CI: 2.93–3.69) and >90 years (chi² = 4.80, *p* = 0.028, OR = 1.83, CI: 1.06–3.17). While less women than men lived alone until the age of 50, they did so significantly more frequently in age 50 and up (Figure 1, middle).

### 3.2. Pre-Stroke Disability

Between >70–≤90 years of age, women had a significantly higher degree of pre-stroke disability than men (Z = −10.18, *p* < 0.001); no differences were found in other age groups (Figure 1, right).

### 3.3. Risk Factors

Except for hypertension, which was more frequently found in women >70–≤90 years, diabetes, hyperlipidemia, and smoking were more prevalent in men in this age segment. Further significant associations between male sex and hyperlipidemia and smoking were identified in ages >30–≤50 years and >50–≤70 years, respectively (Table 1).

### 3.4. Cerebral and Cardiac Vascular Premorbidity

Atrial fibrillation (AF) was significantly more prevalent in women ages >70–≤90 years, all other manifestations of premorbidity (stroke, heart attack, coronary heart disease) were more frequently found in men, as was vascular morbidity in younger patients if present (Table 1).

### 3.5. Stroke Etiology

The proportion of women with cardiac embolism as a cause of the stroke was significantly higher in the age segment >70–≤90 years. In addition, small vessel disease and atherosclerosis were significantly more frequent in men in this age group. Finally, in women aged >30–≤50 years, other etiologies were more frequently identified than in men (Table 2).

## 4. Discussion

Our study of over 11,000 individuals with acute ischemic stroke identified the age segment > 70 years as an important demarcator of change in several characteristics pertinent to ischemic stroke. In addition to confirming the previously identified and roughly U-shaped course of hazard risk for stroke [12] with an upswing in women ages 70 and older, we also found elderly women with stroke to present with more severe pre-stroke disability. As regards vascular premorbidity, AF was more prevalent in women than in men, whereas prior stroke, heart attack, or coronary disease were more frequently found in men. This finding is in line with previous data of a greater risk for stroke in women than in men with AF and general risk increase after the age of 65 [13]. It is also concordant with our observation of significantly more frequent cases of a cardiac source as stroke etiology in women between 70 and 90 years of age.

The sex-difference in pre-stroke disability hence does not appear to be driven by cerebral or cardiac vascular premorbidity, but other conditions such as frailty where a considerable sex gap has been previously noted [14], with significantly more frequent disabling—but non-fatal—comorbidities in women than in men [15]. Moreover, due to their longer life expectancy, women are more likely to live alone, and social isolation, in turn, may yield additional adverse socio-behavioral and medical sequelae [16]. Financial hardship associated with widowhood further contributes to this adversity so that elder female orphans [17]—women without caregivers or family members assisting them in day-to-day affairs—are often unable to afford services needed to live in a comfortable manner and to adequately address their mental and physical health concerns. Other types of comorbidity such as a co-existent psychiatric disorder, may also contribute to higher degrees of actual or perceived social isolation. Notably, there is a distinct female predominance in depression prevalence [18], and associations between pre-existing depressive disorder as well as other psychiatric comorbidities with short- and long-term post-stroke outcomes [19,20] have been noted earlier, thus identifying another important area in need for interdisciplinary preventative strategies. Finally, previous studies found an association between living alone, risk of stroke [21] as well as a delayed presentation with symptoms of stroke [22]. This issue is even more complex when taking into account that women more often than men present with atypical clinical symptoms and signs [23] as well as with stroke mimics [24], which may impede straightforward diagnosis and treatment.

Among the risk factors recorded in our cohort, hypertension is the only one more frequent in women in the age segment >70–≤90 years. In light of the increasing prevalence of this disorder in women of advanced age [25] and recent work implicating that hypertension is associated with greater stroke risk in women than in men [26], effective treatment and control of hypertension is an essential component for stroke prevention in elderly women. A recent study identified important differences between young and older women regarding risk and lifestyle factors associated with stroke incidence and post-stroke functional impairment, necessitating age-dependent differential approaches in secondary and tertiary stroke prevention [27]. With all other vascular risk factors more frequently found in men aged >70–≤90 years, it is not surprising that strokes caused by large and small vessel disease show a distinct male predominance.

The changing patterns of certain sociodemographic and biomedical characteristics pertaining to sex differences in stroke should function as a call to action to improve prevention efforts targeting not only younger [28], but in particular elderly women in their seventies and eighties through targeted education and advice on age-appropriate manageable and sustainable lifestyle modifications as well as the included consideration of living environment and circumstances when it comes to the medical treatment of risk-increasing conditions. For example, technological advances in the last decade or so allow the more widespread utilization of telecare, which has been shown to positively impact on health-related quality of life measures of elder people living on their own [29]. Areas of potential benefit include devices to promote communication with family and friends, emergency assistance, and physical and mental well-being [30].

Limitations of our study are predominantly tied to its retrospective design where comprehensiveness and quality of the source data are of paramount importance. However, as a certified stroke unit, the stroke unit of University Medical Centre, Mannheim, Germany has to meet certain standards regarding the completeness of data required by the German Stroke Society [31]. More detailed information about the social situation, living circumstances, and, importantly, patients’ perception of living alone would be desirable for future analyses. The latter point is relevant in that social isolation and loneliness differentially impact older-age mortality [32].

## 5. Conclusions

Our study highlights the relevance of the interplay of sex and age regarding certain pre-stroke characteristics such as comorbidity and the level of pre-stroke functioning, identifies the seventh and eighth decades as a critical time period in which these features undergo changes shaping the impact of stroke morbidity in women. It emphasizes the relevance of and the need for an approach to stroke prevention that is both targeted and holistic and contributes to the delivery of care in the spirit of 4P (personalized, predictive, preventative, participatory) medicine.

## Figures and Tables

**Table 1:** Results for risk factors and cerebral and cardiac vascular disease premorbidity in men and women separated by age, *n* = 11,003.

	≤30			>30 and ≤50			>50 and ≤70			>70 and ≤90			>90		
	Men	Women	*p*-Value [OR]	Men	Women	*p*-Value [OR]	Men	Women	*p*-Value [OR]	Men	Women	*p*-Value [OR]	Men	Women	*p*-Value [OR]
Risk Factor															
Hypertension, *n* (%)	3 (10.3)	3 (5.0)	0.387 [0.46]	229 (44.6)	116 (39.3)	0.141 [0.80]	1945 (76.7)	897 (78.2)	0.303 [1.09]	2570 (86.0)	2741 (88.8)	0.001 ** [1.30]	68 (87.2)	237 (87.8)	0.888 [1.06]
Diabetes, *n* (%)	0 (0.0)	1 (1.7)	1.000 [1.02]	73 (14.2)	30 (10.2)	0.096 [0.68]	766 (30.2)	322 (28.1)	0.192 [0.90]	1021 (34.2)	971 (31.5)	0.026 * [0.88]	17 (21.8)	56 (20.7)	0.840 [0.94]
Hyperlipidemia, *n* (%)	4 (13.8)	4 (6.7)	0.430 [0.45]	150 (29.2)	50 (16.9)	<0.001 *** [0.49]	931 (36.7)	427 (37.2)	0.757 [1.02]	1060 (35.5)	1004 (32.5)	0.016 * [0.88]	18 (23.1)	59 (21.9)	0.818 [0.93]
Smoking, *n* (%)	10 (34.5)	18 (30.0)	0.669 [0.81]	225 (43.9)	119 (40.3)	0.330 [0.86]	790 (31.1)	298 (26.0)	0.001 ** [0.78]	239 (8.0)	131 (4.2)	<0.001 *** [0.51]	1 (1.3)	3 (1.1)	1.000 [0.86]
Premorbidity															
Stroke, *n* (%)	1 (3.4)	4 (6.7)	1.000 [2.00]	45 (8.8)	20 (6.8)	0.316 [0.76]	418 (16.5)	198 (17.3)	0.554 [1.06]	725 (24.3)	658 (21.3)	0.007 ** [0.85]	14 (17.9)	65 (24.1)	0.255 [1.45]
Heart Attack, *n* (%)	1 (3.4)	0 (0.0)	0.326 [0.97]	19 (3.7)	5 (1.7)	0.105 [0.45]	235 (9.3)	48 (4.2)	<0.001 *** [0.43]	365 (12.2)	204 (6.6)	<0.001 *** [0.51]	8 (10.3)	21 (7.8)	0.485 [0.74]
Atrial Fibrillation, *n* (%)	0 (0.0)	1 (1.7)	1.000 [1.02]	17 (3.3)	7 (2.4)	0.448 [0.71]	336 (13.2)	156 (13.6)	0.768 [1.03]	927 (31.0)	1211 (39.3)	<0.001 *** [1.44]	45 (57.7)	149 (55.2)	0.695 [0.90]
Coronary Heart Disease, *n* (%)	0 (0.0)	0 (0.0)	-	25 (4.9)	3 (1.0)	0.004 ** [0.20]	328 (12.9)	88 (7.7)	<0.001 *** [0.56]	636 (21.3)	376 (12.2)	<0.001 *** [0.51]	12 (15.4)	33 (12.2)	0.463 [0.77]

*: *p* < 0.05, **: *p* < 0.01, ***: *p* < 0.001. Bold print indicates statistical significance.

**Table 2:** Results for stroke etiology in men and women separated by age, *n* = 11,003.

	≤30			>30 and ≤50			>50 and ≤70			>70 and ≤90			>90		
	Men	Women	*p*-Value [OR]	Men	Women	*p*-Value [OR]	Men	Women	*p*-Value [OR]	Men	Women	*p*-Value [OR]	Men	Women	*p*-Value [OR]
Stroke Etiology															
Small Vessel Disease, *n* (%)	3 (10.3)	5 (8.3)	0.712 [0.79]	113 (22.0)	64 (21.7)	0.912 [0.98]	668 (26.3)	283 (24.7)	0.287 [0.92]	566 (18.9)	518 (16.8)	0.029 * [0.86]	7 (9.0)	25 (9.3)	0.939 [1.03]
Atherosclerosis, *n* (%)	3 (10.3)	8 (13.3)	1.000 [1.33]	99 (19.3)	54 (18.3)	0.729 [0.94]	517 (20.4)	221 (19.3)	0.435 [0.93]	564 (18.9)	380 (12.3)	<0.001 *** [0.60]	3 (3.8)	19 (7.0)	0.431 [1.89]
Cardiac Source, *n* (%)	4 (13.8)	12 (20.0)	0.475 [1.56]	77 (15.0)	35 (11.9)	0.213 [0.76]	558 (22.0)	245 (21.4)	0.666 [0.96]	1057 (35.4)	1347 (43.7)	<0.001 *** [1.42]	48 (61.5)	160 (59.3)	0.718 [0.91]
ESUS, *n* (%)	6 (20.7)	15 (25.0)	0.654 [1.28]	60 (11.7)	36 (12.2)	0.830 [1.05]	280 (11.0)	139 (12.1)	0.338 [1.11]	281 (9.4)	295 (9.6)	0.830 [1.02]	6 (7.7)	21 (7.8)	0.980 [1.01]
Other, *n* (%)	7 (24.1)	9 (15.0)	0.293 [0.55]	59 (11.5)	52 (17.6)	0.015 * [1.65]	140 (5.5)	76 (6.6)	0.185 [1.21]	115 (3.8)	103 (3.3)	0.287 [0.86]	1 (1.3)	3 (1.1)	1.000 [0.86]
Unidentified Cause, *n* (%)	6 (20.7)	11 (18.3)	0.791 [0.86]	105 (20.5)	54 (18.3)	0.457 [0.87]	374 (14.7)	183 (16.0)	0.341 [1.10]	406 (13.6)	442 (14.3)	0.403 [1.06]	13 (16.7)	42 (15.6)	0.813 [0.92]

>ESUS, embolic stroke of unknown source; *: *p* < 0.05, ***: *p* < 0.001. Bold print indicates statistical significance.

## Data Availability

The data are available upon reasonable request from the senior author of the study.
